# Tissue Factor and Its Cerebrospinal Fluid Protein Profiles in Parkinson’s Disease

**DOI:** 10.3233/JPD-240115

**Published:** 2024-10-15

**Authors:** Milan Zimmermann, Madeleine Fandrich, Meike Jakobi, Benjamin Röben, Isabel Wurster, Stefanie Lerche, Claudia Schulte, Shahrzad Zimmermann, Christian Deuschle, Nicole Schneiderhan-Marra, Thomas O. Joos, Thomas Gasser, Kathrin Brockmann

**Affiliations:** a Center of Neurology, Department of Neurodegeneration and Hertie-Institute for Clinical Brain Research, University of Tuebingen, Tübingen, Germany; b German Center for Neurodegenerative Diseases (DZNE), University of Tuebingen, Tübingen, Germany; c NMI Natural and Medical Sciences Institute at the University of Tuebingen, Reutlingen, Germany

**Keywords:** Tissue factor, neurodegenerative diseases, blood coagulation, aging, Parkinson’s disease

## Abstract

**Background:**

Prior investigations have elucidated pathophysiological interactions involving blood coagulation and neurodegenerative diseases. These interactions pertain to age-related effects and a mild platelet antiaggregant function of exogenous *α*-Synuclein.

**Objective:**

Our study sought to explore whether cerebrospinal fluid (CSF) levels of tissue factor (TF), the initiator of the extrinsic pathway of hemostasis, differ between controls (CON) compared to patients with Parkinson’s disease (PD) and dementia with Lewy bodies (DLB), considering that these conditions represent a spectrum of *α*-Synuclein pathology. We further investigated whether TF levels are associated with longitudinal progression in PD.

**Methods:**

We examined CSF levels of TF in 479 PD patients, 67 patients diagnosed with DLB, and 16 CON in order to evaluate potential continuum patterns among DLB, PD, and CON. Of the 479 PD patients, 96 carried a *GBA1* variant (PD *GBA1*), while the 383 non-carriers were classified as PD wildtype (PD *WT*). We considered both longitudinal clinical data as well as CSF measurements of common neurodegenerative markers (amyloid-β 1-42, h-Tau, p-Tau, NfL, *α*-Synuclein). Kaplan-Meier survival and Cox regression analysis stratified by TF tertile levels was conducted.

**Results:**

Higher CSF levels of TF were associated with an older age at examination in PD and a significant later onset of postural instability in PD *GBA1*. TF levels were lower in male vs. female PD. DLB *GBA1* exhibited the lowest TF levels, followed by PD *GBA1*, with CON showing the highest levels.

**Conclusions:**

TF as representative of blood hemostasis could be an interesting CSF candidate to further explore in PD and DLB.

## Plain Language Summary

Parkinson’s disease is a common age-related condition, primarily affecting older individuals. However, it shows a wide range of symptoms and clinical courses, influenced by genetic mutations, neuroinflammatory processes and lifestyle factors. Research into the disease’s mechanisms is important for developing new therapies that could potentially slow its progression. Early diagnosis is also essential, as new disease-modifying therapies are most effective when started at an early stage of the disease. In this paper, we focus on a protein called tissue factor, which plays a role in both blood coagulation and neuroinflammation. Proteins involved in blood-coagulation also exhibit an increase in blood-concentration with higher age. Also, a subtle platelet antiaggregant function of exogenous *α*-Synuclein was found, a protein which aggregates in the brain of patients with Parkinson’s disease. Additionally, higher tissue factor levels were found in plaques of proteins (amyloid-β 1-42) found in Alzheimer’s disease. Thus, tissue factor could be a promising biomarker candidate for neurodegenerative diseases. We analyzed the concentration of this protein in the cerebrospinal fluid of 479 patients with Parkinson’s disease, 16 control participants, and 67 patients with dementia with Lewy bodies, a sub-type of Parkinson’s disease with exceptionally high levels of *α*-Synuclein in the brain. Our findings showed the lowest levels of tissue factor in patients with dementia with Lewy bodies, followed by those with typical Parkinson’s disease, and the highest levels in controls. Additionally, older patients had higher tissue factor levels than younger patients, and levels were lower in male patients compared to female patients. Thus, measuring tissue factor levels could help in diagnosing Parkinson’s disease. Further studies, especially with larger control groups, are needed to confirm these results. Additionally, exploring the connections between blood coagulation and Parkinson’s disease could improve our understanding of the disease’s mechanisms, potentially leading to new pharmaceutical developments.

## INTRODUCTION

Parkinson’s disease (PD) stands as a paradigm for age-related disorders, as its incidence escalates with advancing age, highlighting age as a predominant risk factor for PD.^1^ Additionally, outcomes from community-based longitudinal cohorts consistently demonstrate that chronological age and an elevated age at disease onset independently forecast cognitive decline, deterioration in motor function and heightened mortality in individuals with PD.^2–4^

Neuroinflammation plays a crucial role in both the onset and progression of PD. In this context, the cytokines interleukin-1 beta (IL-1β), interleukin-6 (IL-6), monocyte chemoattractant protein 1 (MCP-1), and tumor necrosis factor alpha (TNF- *α*) are consistently highlighted in literature.^5^

It is firmly established that components of blood coagulation, particularly fibrinogen, factor V and factors VII-IX, exhibit an age-related increase. This phenomenon contributes to a procoagulatory condition associated with advanced age.^6^

**Fig. 1:**
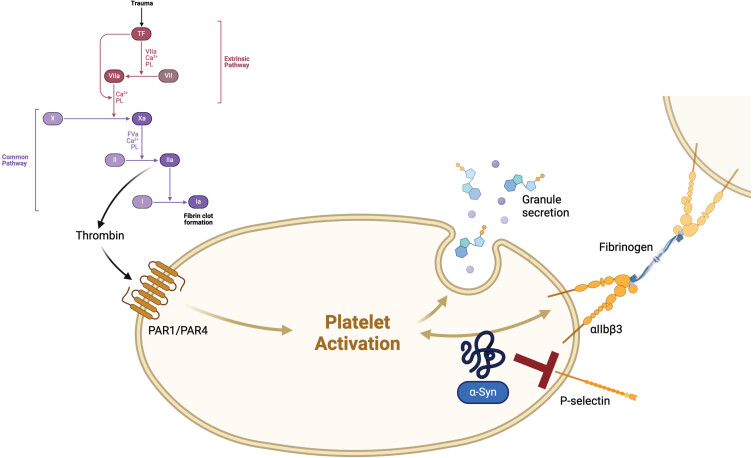
Tissue factor (TF), also known as coagulation factor III, stands at the beginning of the coagulation cascade. An injury of blood vessels leads to the association of a complex with FVIIa, which activates FX and FIX. The complex of FXa and FVa transforms prothrombin (FII) to thrombin (FIIa), which itself leads to an activation of platelets via protease-activated receptors (PARs) and fibrin-cross-linkage via FXIIIa, causing a clot in the end.^8^ *α*-Synuclein (*α*-Syn) might exceed a mild platelet antiaggregant function physiologically, mostly via inhibition of P-selectin expression on the surface of platelets and thus inhibiting thrombin-induced platelet activation.^15^ Created with BioRender.com

Additionally, these proteins such as fibrinogen and prothrombin are involved in inflammatory processes, serving as acute-phase proteins.^7^ Tissue factor (TF), also known as coagulation factor III, plays a crucial role in initiating the extrinsic pathway of hemostasis. Thrombin, as illustrated in Fig. 1, acts as a downstream component in this cascade.^8^ Cytokines like IL-1, endotoxin and TNF can induce TF expression on monocytes. Conversely, chemokine production, including macrophage inflammatory protein-1 *α* (MIP-1*α*), has been found to be induced following TF injections.^9^ Injections of thrombin in a mouse model resulted in heightened microglial activation, subsequently leading to the degeneration of nigral dopaminergic neurons.^10^

Therefore, TF and also thrombin might be promising links between coagulation, (neuro-) inflammation and neurodegeneration.

Growing evidence reveals other potential connections between blood coagulation and PD. Accumulation of *α*-Synuclein in the central and peripheral nervous system, referred to as Lewy bodies, is a pathophysiological hallmark in both PD and dementia with Lewy bodies (DLB).^11^ Within this framework, *α*-Synuclein aggregates serve as damage-associated molecular patterns (DAMPs), triggering microglial activation and cytokine production.^12,13^ Recently, a disease spectrum between PD, PD with dementia (PDD), and DLB based on *α*-Synuclein deposition was proposed, with the most pronounced Lewy body pathology in DLB patients,^14^ especially when exhibiting variants in *GBA1.* ^11^ In the context of blood coagulation, a subtle platelet antiaggregant function of exogenous *α*-Synuclein, which is also present in platelets, has been elucidated. This physiological function of *α*-Synuclein is primarily attributed to the inhibition of P-selectin expression on the platelet surface, thereby impeding thrombin-induced platelet activation.^15^ In animal models, P-selectin has been found to increase TF biosynthesis.^16^

Notably, heightened immunoreactivity for tissue factor antigen^17^ and an increased expression of thrombin have been observed within amyloid-β 1–42 plaques in individuals with Alzheimer’s disease (AD).^18^ Given that concurrent AD pathology, alongside additional vascular risk profiles, has been demonstrated to contribute to cognitive decline in PD patients,^19^ it is plausible that these findings may also apply, at least in part, to PD, PDD, and DLB.

Considering these pathophysiological connections, our objective was to investigate TF in PD and DLB through a two-pronged hypothesis:

1. TF levels might reflect disease stages and progression of PD. We analyzed cross-sectional and longitudinal clinical data in a first step. We also considered common neurodegenerative proteins (amyloid-β 1-42, h-Tau, p-Tau, NfL, and *α*-Synuclein), as they reflect different stages of neuronal damage and highlight additional AD pathology. This is particularly relevant because high levels of TF antigen have been found in senile plaques.
2. CSF TF levels might mirror the continuum of Lewy body pathology observed in patients with PD, PDD, and DLB, with the most pronounced *α*-Synuclein deposition in patients with DLB and *GBA1* variants. Therefore, we stratified by *GBA1* mutational status and included DLB as an additional cohort in all analyzes.

## METHODS

### Participants and clinical investigations

All 479 PD patients (referred to as PD *total cohort*) and 67 patients with DLB were recruited and examined between 2001 and 2022 from the ward and outpatient clinic for PD at the University of Tuebingen. All patients were examined by a movement disorder specialist. The diagnosis of PD was defined according to UK Brain Bank criteria.^20^ The following demographic and clinical data were obtained: age, sex, age at onset of parkinsonism. Additionally, we calculated the disease duration. We assessed the severity of motor symptoms using the Unified Parkinson’s Disease Rating scale part III (UPDRS III); from 2000–2008, and from 2009, the MDS-UPDRS were applied.^21^ The clinical diagnosis of DLB was established in accordance with the revised consensus criteria for DLB, as outlined in the fourth report of the DLB consortium.^22^ Cognitive function was tested using the Montreal Cognitive Assessment (MoCA)^23^ or the Mini-Mental Status Examination (MMSE).^24^ As the MoCA has only been available since 2009, all MMSE scores were converted to MoCA equivalent scores according to an algorithm published recently.^25^ MoCA cutoff ≤25 indicated cognitive impairment (point of maximum combined sensitivity and specificity).^26^ Postural instability was characterized using the UPDRS III score, whereby patients demonstrating three or more compensatory steps in the pull test were classified as having postural instability. Depressive symptoms were assessed using Beck’s Depression Inventory (BDI) II.^27^ The PD cohort, subjected to tissue factor measurements, was chosen based on the criterion of possessing a comprehensive clinical data set. Of the 479 PD patients, 297 had longitudinal data pertaining to the onset of postural instability, forming a smaller longitudinal sub-cohort.

All 16 control participants (CON) were spouses of patients with PD or patients with functional disorders in whom neurodegenerative diseases have been extensively excluded on the ward.

All CSF samples were obtained from the Neuro-Biobank of the University of Tuebingen, Germany (https://www.hih-tuebingen.de/en/about-us/core-facilities/biobank/). The biobank is supported by the local University, the Hertie Institute and the DZNE.

All participants gave written informed consent. The study was approved by the Ethics Committee of the Faculty of Medicine at the University of Tuebingen (Project-Nr. 458/2023BO2). All procedures are in accordance with the Declaration of Helsinki (1975).

### Collection of CSF samples

Spinal tap was performed between 9:00 AM and 1:00 PM. Samples were directly taken from the bedside and centrifuged within 60 min after collection and frozen at –80°C within 90 min after collection. Samples with abnormal routine CSF diagnostics (white blood cell count >6 cells/*μ*L; Immunoglobuline subtype G index >0.7) were excluded.

### CSF measurements of amyloid-β 1-42, h-Tau, p181-Tau (p-Tau), neurofilament light protein (NfL) and *α*-Synuclein

CSF levels of amyloid-β 1-42, h-Tau and p-Tau were measured using ELISA kits from INNOTEST, Fujirebio GmbH, Germany. CSF levels of NFL were measured using the UmanDiagnostics NF- light^®^ assay. Intra-assay coefficients of variation for each CSF parameter were below 15%. CSF levels of total *α*-Synuclein were assessed using an ELISA kit for human *α*-Synuclein (Roboscreen GmbH, Germany). Intra-assay imprecision of 4.4% was calculated from duplicate analyses and expressed as median of the range to average of the duplicates. Inter-assay imprecision of <10% was determined using two quality control CSF pool samples.

All measurements were performed by board-certified laboratory technicians who were blinded to the clinical data.

### Bead-based detection of tissue factor in CSF

Tissue factor CSF levels (in pg/ml) were detected using a commercial 3-plex human magnetic Luminex assay (R&D Systems, Wiesbaden, Germany, cat no. LXSAHM-03, lot number L139408). Individual analyses were performed with 1:2 diluted samples according to the manufacturer’s protocol. For the multiplex assay, 50*μ*L Luminex beads and 50*μ*L standards, quality control or CSF samples were pipetted into the wells of a 96-well plate. The specific analytes are bound by the immobilized antibodies on the color-coded beads. Unbound sample was removed, and beads washed three times with wash buffer. 50*μ*L biotinylated detection antibody was added to each well and incubated at 800 rpm for 1 h at room temperature. The beads were washed three times to remove unbound detection antibody. 50*μ*L Streptavidin-PE conjugate was added to each well. After 30 min incubation at 800 rpm, unbound Streptavidin-PE was removed, and the beads washed three times. The beads were resuspended in 100*μ*l of buffer and then analyzed using the FlexMap 3D^®^ analyser and Luminex xPONENT^®^ 4.2 software (Luminex, Austin, TX, USA). Median fluorescence intensity (MFI) values were back-calculated using a 5PL regression fit to the standard samples (Bio-Plex Manager, version 6) to determine tissue factor concentrations.

### Genetic analysis

DNA was isolated from ethylenediaminetetraacetic acid blood by salting out method and stored at 4°C. Genetic screening for PD-associated mutations was done using the Neurochip. Known pathogenic point mutations in the genes *GBA1*, *LRRK2*, *PRKN*, *PINK1*, and *DJ1* detected by Neurochip were confirmed by Sanger sequencing. Multiplex ligation-dependent probe amplification was used to detect deletions and duplications in the genes *PRKN*, *PINK1* and *DJ1*.

We performed *GBA1*-subgroup classification for mutation severity according to established mutational risk reported for PD: low risk (“risk”, N = 48), mild risk (“mild”, N = 20), severe risk (“severe”, N = 28). Of note, some variants that have been reported as nonrelevant for Gaucher disease have been proven to increase the risk for PD and therefore have been included in our analysis, for example, p. E326K and p.T369M.

### Statistics

Statistical analysis was performed using IBM-SPSS Statistics (IBM Corp. Released 2021. IBM SPSS Statistics for Macintosh, Version 28.0. Armonk, NY: IBM Corp.). Group comparisons were conducted among following groups: PD *GBA1* (N = 96), comprising patients with variants in the *GBA1* gene, PD wildtype (WT, N = 383) (patients without variants in *GBA1*), PD total cohort (PD *GBA1* and PD WT, N = 479), CON (control participants, N = 16) and patients with dementia with Lewy bodies (DLB, N = 67).

The significance level was set at *p*≤0.007 for cross-sectional analysis (manual Bonferroni-correction due to comparison of 7 groups) (Table 1 and Supplementary Table 1), at *p*≤0.016 for comparison of TF levels between CON, PD, and DLB (Supplementary Table 2) and at *p*≤0.05 for longitudinal analysis (Supplementary Table 3).

**Table 1:** Demographic, clinical and CSF-biomarker data in patients with Parkinson’s disease (PD), dementia with Lewy Bodies (DLB), and controls (CON)

	CON	PD			DLB		
		PD total	PD WT	PD *GBA1*	DLB total	DLB WT	DLB *GBA1*
age at examination	65.6 (±9.5)	65.4 (±9.9)	65.9 (±9.9)	63.4 (±9.4)	72.5 (±6.5)	73.6 (±6.4)	69.4 (±6.0)
	N = 16	N = 479	N = 383	N = 96	N = 67^*^	N = 49^*^	N = 18
age at onset		58.1 (±10.5)	58.8 (±10.5)	55.1 (±10.2)	69.3 (±7.3)	70.6 (±7.3)	66.1 (±6.2)
		N = 479	N = 383	N = 96	N = 66	N = 48	N = 18
disease duration		7.3 (±5.2)	7.1 (±5.0)	8.3 (±5.6)	3.2 (±2.1)	3.1 (±2.2)	3.4 (±1.8)
		N = 479	N = 383	N = 96	N = 66	N = 48	N = 18
UPDRS III	1.3 (±2.6)	26.1 (±11.5)	26.0 (±11.5)	26.6 (±11.7)	26.8 (±9.2)	25.5 (±7.9)	28.0 (±10.4)
	N = 7	N = 446^§§^	N = 354^§§^	N = 92^§§^	N = 21^**^	N = 10^**^	N = 11^**^
MoCA	27.7 (±2.2)	25.3 (±3.9)	25.4 (±3.6)	24.8 (±4.8)	15.1 (±4.3)	14.6 (±4.0)	16.0 (±4.7)
	N = 11	N = 403	N = 315	N = 88	N = 43^**^	N = 28^**^	N = 15^**^
BDI II	4.5 (±6.2)	9.1 (±7.2)	8.9 (±7.1)	10.2 (±7.3)	10.5 (±6.5)	12.0 (±6.0)	3.0
	N = 10	N = 348	N = 275	N = 73^§^	N = 6	N = 5	N = 1
Amyloid-β 1–42	984.1 (±233.7)	719.9 (±269.4)	716.8 (±268.8)	732.2 (±272.9)	487.8 (±218.4)	455.5 (±219.7)	573.8 (±195.5)
(in pg/ml)	N = 14	N = 457^§§^	N = 364^§§^	N = 93^§§^	N = 66^**^	N = 48^**^	N = 18^**^
h-Tau	270.6 (±79.6)	249.7 (±130.5)	250.0 (±129.6)	248.8 (±134.7)	320.9 (±222.7)	356.7 (±242.0)	225.4 (±120.3)
(in pg/ml)	N = 14	N = 457	N = 364	N = 93	N = 66	N = 48	N = 18
p-Tau	48.9 (±14.8)	42.2 (±17.0)	42.6 (±17.0)	40.6 (±16.8)	46.0 (±25.8)	49.4 (±27.6)	36.9 (±17.8)
(in pg/ml)	N = 14	N = 446	N = 355	N = 91	N = 62	N = 45	N = 17
NfL	776.5 (±423.2)	969.6 (±797.6)	983.9 (±840.5)	914.6 (±605.6)	1709.9 (±1711.2)	1843.5 (±1927.3)	1372.0 (±937.0)
(in pg/ml)	N = 10	N = 437	N = 347	N = 90	N = 60^*^	N = 43^*^	N = 17
*α*-Synuclein	652.0 (±237.9)	614.2 (±298.0)	630.9 (±306.3)	545.2 (±250.9)	515.5 (±302.0)	531.9 (±313.5)	471.9 (±273.2)
(in pg/ml)	N = 11	N = 451	N = 363	N = 88	N = 62	N = 45	N = 17
tissue factor	534.4 (±161.6)	454.0 (±155.9)	457.3 (±160.1)	440.7 (±137.8)	450.4 (±182.1)	469.7 (±200.3)	398.0 (±107.3)
(in pg/ml)	N = 16	N = 479	N = 383	N = 96	N = 67	N = 49	N = 18^*^

Non-parametric Mann-Whitney-U test with significant *p*-values ≤0.007 (manual Bonferroni-correction) presented as following: § PD vs. CON; * DLB vs. CON; 0.001 < *p*≤0.007: §/*; *p*≤0.001: §§/**. WT, wildtype; *GBA1*, variant in the gene for glucocerebrosidase 1; UPDRS III, Unified Parkinson’s Disease Rating Scale part III; MoCA, Montreal Cognitive Assessment; BDI II, Beck’s Depression Inventory II; NfL, neurofilament light chain.

### Cross-sectional analysis

Group comparisons for continuous variables of demographic, clinical and CSF data between DLB, PD, and CON as well as between *GBA1* subgroups were conducted using either the non-parametric Mann-Whitney U test (Table 1; due to limited number of CON participants) or parametric analysis of covariance for all other analysis (ANCOVA), with covariates sex, age at examination and disease duration as appropriate (for comparison of TF levels between male and female and for *GBA1*-subgroup analysis).

Group comparisons of categorical variables (such as the incidence of cognitive impairment and postural instability; prevalence of intake of blood-thinning medication; prevalence of *GBA1* variants) were conducted using the chi-squared test.

Pearson correlation analysis was employed to investigate the influence of TF levels and age at examination on demographic, clinical and biomarker data (Supplementary Table 1).

### Analysis of TF levels

Comparison of CSF TF levels between PD, DLB, and CON was performed using non-parametric Kruskal-Wallis test, followed by post-hoc tests (Supplementary Table 2).

### Longitudinal analysis

Kaplan-Meier curves, along with Cox regression analysis incorporating the factor “tertile group”, were employed for longitudinal analysis. For these analyses, we compared the lowest vs. mid vs. highest tertile of CSF TF levels (Supplementary Table 3).

## RESULTS

### Demographic and cross-sectional data

#### PD total cohort

Of the 479 PD patients, 313 were male (65.3%). The mean age at examination was 65.4 years (±9.9), the mean age at onset 58.1 years (±10.5), the mean disease duration 7.3 years (±5.2), the mean MoCA score 25.3 (±3.9), the mean UPDRS III score 26.1 (±11.5) and the mean BDI II score 9.1 (±7.2) (Table 1). There was no distinction between male and female PD patients concerning the age at examination (65.4 (±9.9) vs. 64.9 (±10.2), *p* = 0.102).

Within the PD patient cohort, there were 59 individuals (12.3%) using acetylsalicylic acid alone, 4 individuals (0.8%) using a combination of acetylsalicylic acid and clopidogrel, 2 individuals (0.4%) using a combination of acetylsalicylic acid and ticagrelor, 1 individual (0.2%) using clopidogrel alone and 1 individual (0.2%) using rivaroxaban (a direct factor X inhibitor). We observed a heightened prevalence of blood-thinning medication intake, including inhibitors of platelet aggregation or anticoagulants, in PD patients with the highest levels of TF (lowest tertile: 17/133 (12.8%), mid tertile: 16/139 (11.5%), highest tertile: 34/140 (24.3%), *p* = 0.006).

Higher TF levels in CSF were associated with a higher age at examination (r (correlation coefficient according to Pearson) = 0.292, *p*≤0.001), a higher age at onset (*r* = 0.221, *p*≤0.001) and higher CSF levels of amyloid-β 1–42 (*r* = 0.250, *p*≤0.001), h-Tau (*r* = 0.513, *p*≤0.001), p-Tau (*r* = 0.603, *p*≤0.001), NfL (*r* = 0.213, *p*≤0.001), and *α*-Synuclein (*r* = 0.538, *p*≤0.001).

A higher age at examination was associated with a higher age at onset, a longer disease duration, a lower MoCA score, a higher UPDRS III score and higher CSF levels of h-Tau, p-Tau, NfL, and *α*-Synuclein (Supplementary Table 1).

Male PD patients demonstrated lower CSF levels of TF compared to female patients (436.8 (±142.4) vs. 486.5 (±174.3), *p* = 0.003).

### PD WT

383 out of 479 patients had no variant in the *GBA1* gene, referred to as PD WT. 65.0% (249) of those patients were male. The mean age at examination was 65.9 years (±9.9), the mean age at onset 58.8 years (±10.5), the mean disease duration 7.1 years (±5.0), the mean MoCA score 25.4 (±3.6), the mean UPDRS III score 26.0 (±11.5) and the mean BDI II score 8.9 (±7.1) (Table 1).

There was no significant difference between male versus female PD WT regarding the age at examination (65.5 (±10.2) vs. 66.7 (±9.4), *p* = 0.268).

We observed an increased prevalence of blood-thinning medication intake in PD WT who exhibited the highest levels of TF (lowest tertile: 12/103 (11.7%), mid tertile: 12/110 (10.9%), highest tertile: 28/110 (25.5%), *p* = 0.004).

Higher TF levels in CSF were associated with a higher age at examination (*r* = 0.328, *p*≤0.001), a higher age at onset (*r* = 0.256, *p*≤0.001) and higher CSF levels of amyloid-β 1–42 (*r* = 0.233, *p*≤0.001), h-Tau (*r* = 0.548, *p*≤0.001), p-Tau (*r* = 0.613, *p*≤0.001), NfL (*r* = 0.207, *p*≤0.001), and *α*-Synuclein (*r* = 0.540, *p*≤0.001) (Supplementary Table 1).

Male PD patients demonstrated significantly lower CSF levels of TF compared to female patients (436.5 (±142.7) vs. 496.0 (±182.4), *p*≤0.001).

### PD GBA1

Among the 479 patients with PD, 96 patients had a genetic variant in the gene for glucocerebrosidase 1 (referred to as PD *GBA1*). In PD *GBA1*, 64 out of 96 patients were male (66.7%). The mean age at examination was 63.4 years (±9.4), the mean age at onset 55.1 years (±10.2), the mean disease duration 8.3 years (±5.6), the mean MoCA score 24.8 (±4.8), the mean UPDRS III score 26.6 (±11.7) and the mean BDI II score 10.2 (±7.3) (Table 1).

There was no significant difference between male versus female PD *GBA1* regarding the age at examination (62.4 (±9.7) vs. 65.4 (±8.7), *p* = 0.154).

There was no significant difference observed in terms of the use of blood-thinning medication (*p* = 0.507) or the prevalence of severe *GBA1* variants (*p* = 0.078) among the CSF TF tertiles.

Higher TF levels in CSF were associated with higher CSF levels of amyloid-β 1–42 (*r* = 0.331, *p*≤0.001), h-Tau (*r* = 0.371, *p*≤0.001), p-Tau (*r* = 0.557, *p*≤0.001), and *α*-Synuclein (*r* = 0.520, *p*≤0.001) (Supplementary Table 1).

No significant difference was observed between male and female patients regarding CSF levels of TF (*p* = 0.931). Furthermore, no variance was identified in CSF TF levels based on the severity of *GBA1* variants (risk: 438.3 (±142.6), mild: 476.5 (±109.6), severe: 419.5 (±146.6), *p* = 0.474).

### DLB cohort

Out of 67 patients diagnosed with DLB, 46 were male, constituting a prevalence of 68.7%. The mean age at examination was 72.5 years (±6.5), which is notably higher than the age at examination in CON (*p*≤0.001) and PD (*p*≤0.001). The mean age at onset was 69.3 years (±7.3), the mean disease duration 3.2 years (±2.1), the mean MoCA score 15.1 (±4.3), the mean UPDRS III score 26.8 (±9.2) and the mean BDI II score 10.5 (±6.5). 18 patients had a variant in *GBA1* (26.9%) (Table 1). 88.9% (16/18) of these patients were male.

Higher TF levels in CSF were associated with higher CSF levels of h-Tau (*r* = 0.689, *p*≤0.001), p-Tau (*r* = 0.812, *p*≤0.001), and *α*-Synuclein (*r* = 0.750, *p*≤0.001) (Supplementary Table 1).

No statistically significant differences were observed between males and females with DLB regarding the CSF TF levels (*p* = 0.766).

### CON

Of the 16 control participants (CON), 7 were male (43.8%). The mean age at examination was 65.6 years (±9.5), the mean MoCA score 27.7 (±2.2), the mean UPDRS III score 1.3 (±2.6) and the mean BDI II score 4.5 (±6.2) (Table 1).

There were no significant differences concerning the age at examination between CON vs. PD total cohort (*p* = 0.935), CON vs. PD WT (*p* = 0.794) or CON vs. PD *GBA1* (*p* = 0.549).

Higher TF levels in CSF were associated with higher CSF levels of h-Tau (*r* = 0.844, *p*≤0.001) and p-Tau (*r* = 0.786, *p*≤0.001) (Supplementary Table 1).

### Group comparison of TF levels between CON, PD, and DLB

There was a significant difference between the three cohorts regarding CSF TF levels with the lowest levels in DLB *GBA1* (398.0 pg/ml (±107.3)), followed by PD *GBA1* (440.7 pg/ml (±137.8)) and CON (534.4 pg/ml (±161.6)) (*p* = 0.034). *Post-hoc* tests revealed a significant difference in TF levels between DLB *GBA1* vs. CON (*p* = 0.012; PD *GBA1* vs. CON: *p* = 0.030; PD *GBA1* vs. DLB *GBA1*: *p* = 0.271) (Supplementary Table 2).

### Longitudinal analysis

#### PD total cohort

The follow-up duration (in years) revealed no statistically significant differences among the three tertiles of CSF TF levels (*p* = 0.337).

Likewise, the incidence of cognitive impairment (*p* = 0.712) and postural instability (*p* = 0.976) did not demonstrate statistically significant variations across the three tertile groups throughout the longitudinal study.

**Fig. 2:**
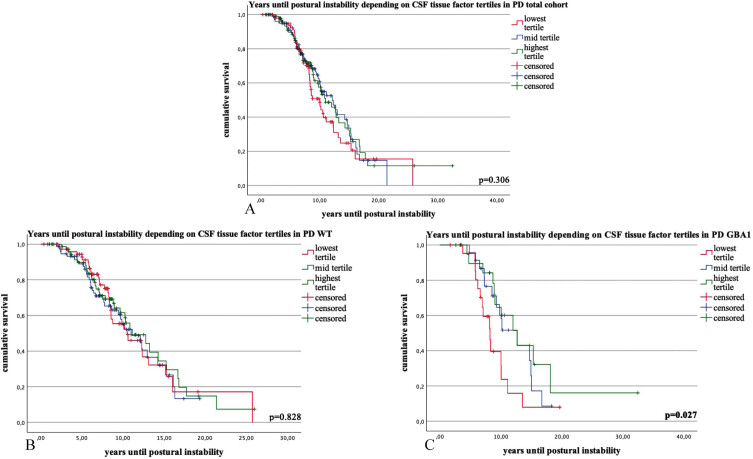
Kaplan-Meier survival curves and COX regression analysis for the time interval (in years) until 50% of the Parkinson’s disease (PD) patients reached the milestone postural instability (PI) in PD total cohort (1A), PD wildtype (1B) and PD *GBA1* (1C), stratified by tertiles of CSF tissue factor (TF) levels. Categories: lowest tertile of tissue factor CSF levels; mid tertile of tissue factor levels; highest tertile of tissue factor levels. *p*-values ≤0.05 are highlighted in bold.

The time interval until 50% of patients reached postural instability did not exhibit any statistically significant differences based on the TF tertiles (*p* = 0.306) (Fig. 2 and Supplementary Tables 3 and 4).

### PD WT

Patients with the highest TF tertiles exhibited statistically significant longer follow-up times (lowest tertile: 7.9 (±4.3), N = 77; mid tertile: 8.1 (±3.9), N = 76; highest tertile: 8.2 (±4.7), N = 76; *p* = 0.047).

Throughout the follow-up times, there were no discernible differences in the incidence of postural instability (*p* = 0.769) and cognitive impairment (*p* = 0.130) among the three tertile groups. No statistically significant differences were observed in the time interval until 50% of patients reached postural instability based on the TF tertiles (*p* = 0.828) (Fig. 2 and Supplementary Tables 3 and 4).

### PD GBA1

There were longer follow-up times in patients with the highest TF tertile (lowest tertile: 7.7 (±3.7), mid tertile: 9.9 (±3.8), highest tertile: 10.5 (±6.5), *p* = 0.033).

The time interval (in years) until 50% of patients reached postural instability differed significantly among the three tertiles of CSF TF levels. Specifically, in the lowest tertile, it was 9.2 years (95% confidence interval: 7.2–11.1, N = 23), in the mid tertile, it was 11.8 years (9.9–13.7, N = 23) and in the highest tertile, it was 14.9 years (9.8–20.1, N = 22) (*p* = 0.027). There was no significant difference in the age at examination (lowest tertile: 59.6 years (±9.5), mid tertile: 60.8 (±8.8), highest tertile: 64.6 (±10.7), *p* = 0.254).

The analysis yielded non-significant findings for the incidence of cognitive impairment (*p* = 0.261) and postural instability (*p* = 0.567) across the three tertile groups throughout the follow-up period (Fig. 2 and Supplementary Tables 3 and 4).

## DISCUSSION

Our results support the hypothesis that tissue factor might serve as a novel biomarker for PD and other Lewy body diseases.

As a first step, we analyzed the association between TF levels and clinical data to determine if these levels reflect the progression of PD.

Higher TF levels in CSF were associated with a higher age at examination in PD, along with increased CSF levels of h-Tau, p-Tau, NfL, and *α*-Synuclein. Given that this pattern was similarly observed in the correlation analysis between the age at examination and the clinical and biomarker data, it is plausible to attribute these findings to age-related effects. Former studies also investigated on age-dependent changes of those CSF biomarkers showing an age-dependent increase in Tau,^28^ NfL,^29^ or *α*-Synuclein.^30^ However, an exception arises in the correlation between elevated TF levels and increased amyloid-β 1–42 in CSF. This deviation contrasts with the expected outcome linked to aging progression, wherein there is typically a decline in amyloid-β 1–42 levels, as partially evidenced by previous studies.^31^ Future studies should address the potential association between TF and amyloid-β 1–42 suggested by this finding, particularly considering the elevated deposition of TF antigen within senile plaques.^17^

Additionally, a significantly higher prevalence of blood-thinning medication intake was observed in PD patients with the highest tertile of TF levels. This finding may also be attributed to the older age at examination and the subsequently increased prevalence of neurovascular diseases. Previous studies demonstrated a reduction in circulating tissue factor following the administration of antiplatelet agents in individuals with peripheral arterial disease,^32^ especially with clopidogrel.^33,34^ This calls for further studies investigating interactions between CSF TF levels and blood-thinning medication.

Elevated CSF levels of TF were linked to a significantly delayed onset of postural instability in PD *GBA1*. Remarkably, patients with the highest TF levels exhibited a trend towards a higher age at examination, although this did not achieve statistical significance in the subgroup of patients with Kaplan-Meier data. Those patients with the highest TF levels also had longer follow-up times. It is essential to note that this observation could introduce potential bias, particularly if patients with a more protective disease course continued participation while others became dropouts.

Another limitation of our current study is the use of both the UPDRS III and, since 2009, the MDS-UPDRS III, which has a broader scale.

It is pertinent to acknowledge that the identification of age-related effects in our study serves not merely as a limitation but also as an opportunity. The quantification of TF protein levels could potentially serve as a surrogate marker, depicting (pathological) aging processes. This aligns with the hypothesis of accelerated aging in PD, dementia and other neurodegenerative conditions.

Our findings also revealed sex-specific differences, with male PD patients exhibiting lower CSF TF levels than their female counterparts. Investigations into sex-specific differences in blood coagulation and fibrinolysis have been conducted in various studies, including PD patients. Estrogen is recognized for its role in increasing coagulation proteins,^35^ which might serve as an explanation for sex-differences at least premenopausal, but not for our cohorts of mainly postmenopausal female patients. Other studies also showed sex-specific differences in coagulation proteins. In this line, elevated levels of Factor VIII were detected in the blood of female PD patients.^36^ Another study noted an age-related increase in TF pathway inhibitor and activated Factor VIIa, particularly in female participants.^37^ This calls for further investigations into those sex-specific differences of blood-coagulation factors especially in female PD patients, also considering postmenopausal supplementation of hormones like estrogen.

We found a significant difference between the three cohorts regarding CSF TF levels with the lowest levels in DLB *GBA1*, higher levels in PD *GBA1* and the highest levels in CON.

This finding might mirror the continuum of Lewy body pathology observed in patients with PD and DLB,^14^ with the most pronounced Lewy body pathology in DLB patients, especially when exhibiting variants in *GBA1*.^11^ A proinflammatory condition preceding and/or induced by Lewy bodies might lead to the local deposition of TF, similar to the situation observed in senile plaques in AD, causing reduced levels of TF in the CSF of patients, analogous to the reduced CSF levels of *α*-Synuclein found in PD patients.

In pathological conditions characterized by Lewy body deposition, there may also be a local procoagulatory state, which contrasts with the proposed mild antiaggregant effects of *α*-Synuclein under normal physiological conditions.^15^ The fact that we only found significant results when comparing CON with PD and DLB patients exhibiting *GBA1* variants might be explained by the higher *α*-Synuclein pathology in these groups compared to PD and DLB wild-type patients. Future studies should address this assumption.

The most significant limitation of our study is the small number of control participants. Therefore, our observations highlight the need for further investigations in larger cohorts.

First studies found effects of thrombin-inhibitors in models of PD. In this line, treatment with direct thrombin inhibitors has demonstrated neuroprotective effects, such as the amelioration of motor deficits and reduction of oxidative stress.^18,38^ This treatment resulted in increased Nurr1 expression and reduced thrombin accumulation in the substantia nigra, activating genes associated with tyrosine hydroxylase and vascular monoamine transporter. This led to elevated dopamine levels and improved motor function.^39^ A deficiency of PAR-1 and a treatment with a PAR-1 antagonist were associated with protective effects against 1-methyl-4-phenyl-1,2,3,6-tetrahydropyridine (MPTP)- induced toxicity.^40^ In this line, compensatory mechanisms in response to neurodegeneration, as previously discussed with the increased expression of PAR-1 in astrocytes of PD patients, merit particular consideration.^41^

Therefore, further investigations into the connections between blood coagulation and PD would be of interest.

## Supplementary Material

Supplementary Material

## Data Availability

The pseudonymized data of this study are available from the corresponding author upon reasonable request.
