# Supplementary material for: Tissue Factor and Its Cerebrospinal Fluid Protein Profiles in Parkinson’s Disease
Source: J Parkinsons Dis. 2024 Oct 15;14(7):1405–16. doi: 10.3233/JPD-240115 (PMC11492048; doi:10.3233/JPD-240115)
Supplement: Supplementary Material [file jpd-14-jpd240115-s001.pdf]

Supplementary Material

Tissue Factor and Its Cerebrospinal Fluid Protein Profiles in Parkinson’s Disease

Supplementary Table 1. Correlation analysis (Pearson's correlation) between A) tissue factor levels (TF) in cerebrospinal fluid (CSF) (in pg/ml) along with demographic data, clinical data and CSF biomarker levels (in pg/ml) in patients with Parkinson's disease (PD), dementia with Lewy bodies (DLB), and controls (CON). B) Age at examination is also analyzed in relation to the aforementioned parameters.

		**A. Tissue factor (TF) CSF levels**							**B. Age at examination**						
		TF PD			TF DLB			TF CON	PD			DLB			CON
		total	WT	*GBA1*	total	WT	*GBA1*		total	WT	*GBA1*	total	WT	*GBA1*	
sex	Eta	0.152	0.178	0.030	0.099	0.067	0.036	0.139							
age at examination	R	0.292	0.328	0.098	0.323	0.308	0.217	0.151							
	P	**≤0.001**	**≤0.001**	0.341	0.008	0.031	0.387	0.577							
	N	479	383	96	67	49	18	16							
age at onset	R	0.221	0.256	0.034	0.296	0.306	0.040		0.873	0.880	0.841	0.961	0.957	0.960	
	P	**≤0.001**	**≤0.001**	0.743	0.016	0.035	0.873		**≤0.001**	**≤0.001**	**≤0.001**	**≤0.001**	**≤0.001**	**≤0.001**	
	N	479	383	96	66	48	18		479	383	96	66	48	18	
disease duration	R	0.106	0.112	0.105	0.010	-0.072	0.594		0.128	0.132	0.163	-0.217	-0.265	-0.014	
	P	0.021	0.029	0.310	0.939	0.629	0.009		**0.005**	0.010	0.112	0.080	0.069	0.956	
	N	479	383	96	66	48	18		479	383	96	66	48	18	
MoCA	R	-0.066	-0.142	0.149	-0.116	-0.180	0.186	-0.216	-0.412	-0.441	-0.396	-0.396	-0.446	-0.233	-0.379
	P	0.185	0.012	0.165	0.457	0.360	0.507	0.523	**≤0.001**	**≤0.001**	**≤0.001**	0.009	0.017	0.403	0.250
	N	403	315	88	43	28	15	11	403	315	88	43	28	15	11
UPDRS III	R	0.004	0.022	-0.074	-0.150	-0.211	-0.052	0.336	0.132	0.134	0.142	0.092	0.582	-0.232	0.200
	P	0.940	0.684	0.483	0.517	0.558	0.880	0.461	**0.005**	0.012	0.176	0.692	0.077	0.492	0.668
	N	446	354	92	21	10	11	7	446	354	92	21	10	11	7
BDI II	R	0.085	0.104	0.017	0.346	0.291		0.070	-0.040	0.027	-0.243	0.287	0.588	.	0.150
	P	0.115	0.085	0.886	0.502	0.635		0.847	0.460	0.658	0.038	0.582	0.297		0.680
	N	348	275	73	6	5		10	348	275	73	6	5		10
Amyloid-β 1-42	R	0.250	0.233	0.331	0.136	0.211	0.048	0.538	-0.073	-0.042	-0.185	-0.084	0.000	-0.060	0.385
	P	**≤0.001**	**≤0.001**	**≤0.001**	0.276	0.149	0.851	0.047	0.121	0.427	0.076	0.504	0.999	0.815	0.174
	N	457	364	93	66	48	18	14	457	364	93	66	48	18	14

h-Tau	R	0.513	0.548	0.371	0.689	0.682	0.637	0.844	0.417	0.430	0.377	0.424	0.393	0.340	0.436
	P	**≤0.001**	**≤0.001**	**≤0.001**	**≤0.001**	**≤0.001**	**0.004**	**≤0.001**	**≤0.001**	**≤0.001**	**≤0.001**	**≤0.001**	**0.006**	0.168	0.119
	N	457	364	93	66	48	18	14	457	364	93	66	48	18	14
p-Tau	R	0.603	0.613	0.557	0.812	0.831	0.635	0.786	0.363	0.397	0.210	0.367	0.340	0.290	0.201
	P	**≤0.001**	**≤0.001**	**≤0.001**	**≤0.001**	**≤0.001**	**0.006**	**≤0.001**	**≤0.001**	**≤0.001**	0.046	**0.003**	0.022	0.259	0.491
	N	446	355	91	62	45	17	14	446	355	91	62	45	17	14
NfL	R	0.213	0.207	0.245	0.162	0.146	0.145	0.732	0.322	0.323	0.313	-0.143	-0.220	-0.010	0.055
	P	**≤0.001**	**≤0.001**	0.020	0.215	0.350	0.579	0.016	**≤0.001**	**≤0.001**	**0.003**	0.277	0.157	0.971	0.879
	N	437	347	90	60	43	17	10	437	347	90	60	43	17	10
α-Synuclein	R	0.538	0.540	0.520	0.750	0.791	0.586	0.307	0.309	0.296	0.352	0.385	0.354	0.457	0.526
	P	**≤0.001**	**≤0.001**	**≤0.001**	**≤0.001**	**≤0.001**	0.014	0.359	**≤0.001**	**≤0.001**	**≤0.001**	**0.002**	0.017	0.065	0.097
	N	451	363	88	62	45	17	11	451	363	88	62	45	17	11

Significant p-values ≤0.007 (manual Bonferroni-correction) are highlighted in bold. R, correlation coefficient according to Pearson; Eta, Eta-squared; P, p-value; N, sample size; WT, wildtype; *GBA1*, variant in the gene for glucocerebrosidase 1; UPDRS III, Unified Parkinson's Disease Rating Scale Part III; MoCA, Montreal Cognitive Assessment; BDI II, Beck's Depression Inventory II; NfL, neurofilament light chain.

Supplementary Table 2. Comparison of CSF levels (in pg/ml) of TF between PD (Parkinson's disease), DLB (dementia with Lewy bodies), and CON for total cohort, WT and *GBA1*.

	Overall				Male				Female			
	DLB	PD	CON	p	DLB	PD	CON	p	DLB	PD	CON	p
total cohort	450.4 (±182.1) N=67	454.0 (±155.9) N=479	534.4 (±161.6) N=16	0.076	438.3 (±164.0) N=46	436.8 (±142.4) N=313	559.1 (±214.1) N=7	0.257	477.1 (±218.6) N=21	486.5 (±174.3) N=166	515.2 (±116.9) N=9	0.403
WT	469.7 (±200.3) N=49	457.3 (±160.1) N=383	534.4 (±161.6) N=16	0.134	459.1 (±183.7) N=30	436.5 (±142.7) N=249	559.1 (±214.1) N=7	0.272	486.5 (±228.3) N=19	496.0 (±182.4) N=134	515.2 (±116.9) N=9	0.505
GBA1	398.0 § (±107.3) N=18	440.7 (±137.8) N=96	534.4 (±161.6) N=16	**0.034**	399.3 (±114.1) N=16	437.8 (±142.4) N=64	559.1 (±214.1) N=7	0.187	387.5 (±15.9) N=2	446.6 (±129.9) N=32	515.2 (±116.9) N=9	0.180

Kruskal-Wallis tests with significant p-values ≤0.05 indicated in bold letters. In case of a significant p-value, post-hoc tests were performed with a manual adapted significance level of p≤0.016 according to Bonferroni indicating significant results (PD vs. DLB: *; PD vs. CON: &; DLB vs. CON: §).

Supplementary Table 3. Kaplan-Meier survival analysis and COX regression analysis for the time interval (in years) until 50% of the Parkinson's disease (PD) patients reached postural instability (PI) in PD total cohort, PD wildtype (WT) and PD *GBA1*, stratified by tertiles of CSF tissue factor levels.

	**Years until postural instability**		
	**overall**	**male**	**female**
**total cohort**	lowest tertile: 11.8 (9.8-13.8), (N=99)<br>mid tertile: 12.0 (10.6-13.5), (N=100)<br>highest tertile: 13.2 (10.7-15.6), (N=98)<br>p= 0.306	lowest tertile: 10.6 (9.1-12.0), (N=70)<br>mid tertile: 12.1 (10.4-13.8), (N=71)<br>highest tertile: 11.9 (8.3-15.6), (N=53)<br>p= 0.744	lowest tertile: 12.9 (8.7-17.2), (N=29)<br>mid tertile: 11.3 (9.3-13.4), (N=29)<br>highest tertile: 13.3 (10.9-15.8), (N=45)<br>p= 0.434
**WT**	lowest tertile: 12.6 (10.0-15.1), (N=77)<br>mid tertile: 11.2 (9.7-12.7), (N=76)<br>highest tertile: 12.4 (10.4-14.5), (N=76)<br>p= 0.828	lowest tertile: 11.2 (9.5-13.0), (N=54)<br>mid tertile: 11.0 (9.3-12.8), (N=55)<br>highest tertile: 10.2 (7.6-12.7), (N=38)<br>p= 0.399	lowest tertile: 14.2 (8.9-19.5), (N=23)<br>mid tertile: 11.2 (8.9-13.6), (N=21)<br>highest tertile: 13.9 (11.0-16.9), (N=38)<br>p= 0.498
GBA1	lowest tertile: 9.2 (7.2-11.1), (N=23)<br>mid tertile: 11.8 (9.9-13.7), (N=23)<br>highest tertile: 14.9 (9.8-20.1), (N=22)<br>p= **0.027**	lowest tertile: 8.8 (6.5-11.0), (N=17)<br>mid tertile: 12.2 (10.1-14.4), (N=16)<br>highest tertile: 16.4 (9.0-23.9), (N=14)<br>p= **0.037**	lowest tertile: 10.6 (7.1-14.1), (N=6)<br>mid tertile: 9.4 (7.8-11.1), (N=7)<br>highest tertile: 12.2 (9.8-14.5), (N=8)<br>p= 0.408

Categories: lowest tertile of tissue factor CSF levels; mid tertile of tissue factor levels; highest tertile of tissue factor levels; 95% confidence intervals presented in brackets. P-values ≤0.05 are highlighted in bold. WT, wildtype; *GBA1*, variant in the gene for glucocerebrosidase 1.

Supplementary Table 4. Demographic and clinical characteristics of Parkinson's disease (PD) patients stratified by PD total cohort, PD wildtype (WT) and PD *GBA1*, based on tissue factor tertile levels in the longitudinal sub-cohort.

	**Demographic and clinical data in the longitudinal sub-cohort**				
		**lowest tertile**	**mid tertile**	**highest tertile**	**p**
**follow-up times**	**PD total cohort**	7.9 (±4.1)	8.6 (±4.2)	8.5 (±5.0)	0.337
	**PD WT**	7.9 (±4.3)	8.1 (±3.9)	8.2 (±4.7)	**0.047**
	**PD *GBA1***	7.7 (±3.7)	9.9 (±3.8)	10.5 (±6.5)	**0.033**
**age at examination**	**PD total cohort**	60.3 (±8.9)	63.3 (±10.3)	67.0 (±9.1)	**≤0.001**
	**PD WT**	60.4 (±8.9)	63.5 (±10.4)	68.3 (±8.4)	**≤0.001**
	**PD *GBA1***	59.6 (±9.5)	60.8 (±8.8)	64.6 (±10.7)	0.254
**incidence of postural instability**	**PD total cohort**	48/99 (48.5%)	48/100 (48.0%)	46/98 (46.9%)	0.976
	**PD WT**	32/77 (41.6%)	36/76 (47.4%)	34/76 (44.7%)	0.769
	**PD *GBA1***	15/23 (65.2%)	14/23 (60.9%)	11/22 (50.0%)	0.567
**incidence of cognitive impairment**	**PD total cohort**	37/85 (43.5%)	36/85 (42.4%)	32/85 (37.6%)	0.712
	**PD WT**	28/66 (42.4%)	31/66 (47.0%)	20/66 (30.3%)	0.130
	**PD *GBA1***	11/19 (57.9%)	6/19 (31.6%)	9/19 (47.4%)	0.261

Analysis of covariance (ANCOVA) incorporating age at examination, sex, and disease duration (follow-up times) or sex (age at examination) as covariates. Chi-Squared test employed for categorical variables, including the incidence of postural instability and cognitive impairment throughout the study duration. P-values ≤0.05 are highlighted in bold. *GBA1*, variant in the gene for glucocerebrosidase 1.
